# The interactome of histone deacetylase HDA19 in dark-grown Arabidopsis seedlings

**DOI:** 10.3389/fpls.2023.1296767

**Published:** 2023-11-23

**Authors:** Isabel Cristina Vélez-Bermúdez, Wolfgang Schmidt

**Affiliations:** Institute of Plant and Microbial Biology, Academia Sinica, Taipei, Taiwan

**Keywords:** immunoprecipitation, proteomics, histone acetylation, etiolated hypocotyl, skotomorphogenesis

## Introduction

As a central epigenetic modification, histone acetylation affects the expression of genes with a wide range of functions across all life forms (Shen et al., 2015). Histone acetylation is mediated by histone acetylases and, generally, promotes DNA-templated transcriptional activity. Histone deacetylases (HDAs) reverse this process, leading to an inactive chromatin state and decreased transcriptional activity. Acetylation and deacetylation are central regulatory switches that govern responses to various environmental cues, orchestrating chromatin dynamics and gene activity to modulate the phenotypic readout.

The Arabidopsis genome harbours 18 HDAs, which are organized in three superfamilies comprised of the HDA classes 1-4 (Seto and Yoshida, 2014). HISTONE DEACETYLASE 19 (HDA19) belongs to the class 1 of the Reduced Potassium Dependence3/Histone Deacetylase-1 (RPD3/HDA1) superfamily and is possibly the most intensively studied HDA. HDA19 has reported roles across a broad landscape of processes and was shown to be a crucial player in seed development (Long et al., 2006; Zhou et al., 2020; Chen X. et al., 2023), pathogen response (Zhou et al., 2005), phosphate deficiency (Chen et al., 2015), light signalling (Jing et al., 2020), the regulation of flowering time (Ning et al., 2019), hormone responses (Mehdi et al., 2016; Kim et al., 2019), and floral organ identity genes (Krogan et al., 2012; Bollier et al., 2018; Ning et al., 2019). Similar to other RPD3-type HDACs, HDA19 forms various complexes that play pivotal roles in various stress responses (Feng et al., 2021; Vélez-Bermúdez and Schmidt, 2021), some of which possess yet unexplored stress-specific compositions.

In this Data Report, we provide an immunoprecipitation (IP)-based analysis of the HDA19 interactome in etiolated Arabidopsis seedlings. HDA19 is directly involved in hypocotyl elongation during photomorphogenesis and, based on our findings, also in hypocotyl elongation during skotomorphogenesis (Vélez-Bermúdez and Schmidt, 2021). The exact role of HDA19 in the latter process is still under investigation. For this reason, we conducted an immunoprecipitation experiment that provides new insights regarding the role of HDA19 in skotomorphogenesis and allows for comparisons of HDA19 targets in hypocotyl elongation during skoto- and photomorphogenesis. We believe that the catalogue of HDA19-interacting proteins presented here provides a valuable resource for follow-up research on novel interacting partners of this central protein. The material used for this experiment was derived from hypocotyls of 6-day-old etiolated Arabidopsis seedlings. The dataset contains a total of 6 files, 3 independent biological replicates of each Col-0 (control plants) and *35S::HDA19-GFP* plants.

## Materials and methods

### Plant materials and growth conditions

*Arabidopsis thaliana* Col-0 and the transgenic line *35S::HDA19-GFP* were used in this study. *35S::HDA19-GFP* lines have been described previously (Zhou et al., 2005). Seeds were soaked in 35% bleach for 5 min, washed five times for 5 min with sterile water, and resuspended in 1 mL of sterile water for further use. Seeds were subsequently placed on a growth medium (Estelle and Somerville, 1987; ES medium) containing 5 mM KNO_3_, 2 mM MgSO_4_, 2 mM Ca (NO_3_)_2_, 2.5 mM KH_2_PO_4_, 70 μM H_3_BO_3_, 14 μM MnCl_2_, 1 μM ZnSO_4_, 0.5 μM CuSO_4_, 0.01 μM CoCl_2_, 0.2 μM Na_2_MoO_4_, and 40 μM Fe-EDTA, solidified with 0.4% Gelrite Pure. MES (1 g/L) and 1.5% (w/v) sucrose were added, and the pH was adjusted to 5.5 with KOH. Seeds were stratified on plates for 2 days at 4°C in the dark and grown at 22°C in vertical position in the dark with 70% relative humidity.

### Immunoprecipitation

Experiments were carried out with the μMACS Epitope GFP tag protein isolation kit (MACSmolecular) following the manufacturer’s instructions with minor modifications. Hypocotyls were collected by dissection in dark conditions within ca. 5 minutes per plate (~25 seedlings). Samples from each plate were frozen immediately in liquid nitrogen. A total of 25 plates were used to obtain 0.5 gram of tissue. Hypocotyls were ground with liquid nitrogen and resuspended in 500 μL of extraction buffer (50 mM Tris/HCl, pH 7.5, 150 mM NaCl, 1% Triton X-100, 2X complete protease inhibitor cocktail EDTA-free (ROCHE), 1 mM PMSF, and 50 μM MG132). The samples were incubated on ice for 30 minutes with occasional mixing and centrifuged for 20 minutes at 10,000 x g at 4°C. The supernatants were individually collected in fresh tubes, and 400 μL of each input was added to 50 μl of anti-GFP microbeads and incubated for 1 hour and 30 minutes in a mixer set to 60 rpm at 4°C, while 100 μL of each input was kept to be used for Western blots using anti-GFP as a control. The samples were eluted in 50 μL denaturing elution buffer supplied with the kit.

### S-Trap sample digestion and protein identification

The protocol was conducted as described previously (Chen C. W. et al., 2023). Briefly, 50 μL eluted IP sample was resuspended in 30 µL of lysis buffer (5% SDS (w/v) in 50 mM triethylammonium bicarbonate (TEAB), pH 8.5), transferred to a 1.7 mL tube, sonicated 10 times for 10 sec each, centrifuged at 16,000 g at 4°C for 20 min, and the supernatant was collected. The IP sample protein amount was determined by using a bicinchoninic acid assay (Thermo Fisher Scientific, Waltham, MA). The IP protein digestion was performed in the S-Trap micro column following the manufacturer’s protocol with some modifications. Shortly, 10 μg of protein in lysis buffer was reduced and alkylated using 1.6 μL of 200 mM tris(2-carboxyethyl)phosphine hydrochloride (TCEP) and 1.6 μL of 800 mM 2-chloroacetamide (CAA) at 45°C for 15 min. After alkylation, 3.3 µL of 55.5% (v/v) phosphoric acid (PA) was added, and the pH (~1) was controlled by means of pH paper. After acidification, the sample was mixed with 198 μL of binding buffer (100 mM TEAB in 90% (v/v), MeOH). After gentle vortexing, the sample was loaded onto an S-trap micro column and centrifuged at 4,000 g for 2 min to trap the proteins. The sample was then washed three times in the column with 150 µL of binding buffer and centrifuged at 4,000 g for 2 min each time. An additional centrifugation step (4,000 g for 2 min) was added to fully remove residual binding buffer. The S-trap column was transferred to a fresh 1.7 mL sample tube for the digestion, and 20 µL of protease solution (Lys-C + trypsin, 50 mM TEAB) was added into individual S-traps containing the samples. The cap of each S-trap was loosely closed to limit evaporative loss, and the samples were incubated for 2.5 h at 47°C. The column was removed from the incubator, and 40 µL of of three buffers were added consecutively to the column: 50 mM TEAB, elution buffer 2 (0.2% formic acid in H_2_O) and elution buffer 3 (50% acetonitrile (ACN) in ultrapure water). The column was centrifuged at 4,000 g for 2 min and the elution solution was collected in a new tube, dried by speed vacuum, resuspended in 100 µL of 0.1% formic acid, desalted, and loaded into a C18 Ziptip pipette tip. The elution was dried down under vacuum, the pellet was re-dissolved in 10 µL of 0.1% (v/v) formic acid (FA) with 3% (v/v) acetonitrile (ACN), and the liquid chromatography was performed by injecting 4 µL of sample in the LC-nESI-Q Exactive mass spectrometer model (Thermo Fisher Scientific) coupled with an on-line nanoUHPLC (Dionex UltiMate 3000 Binary RSLCnano). The Acclaim PepMap 100 C18 trap column (75 µm x 2.0 cm, 3 µm, 100 Å, Thermo Scientific) and the Acclaim PepMap RSLC C18 nano LC column (75 µm x 25 cm, 2 µm, 100 Å) were used to deliver solvent and separate tryptic peptides with a linear gradient from 5% to 35% of acetonitrile in 0.1% (v/v) formic acid for 60 min at a flow rate of 300 nl/min. The acquisition cycle for MS data was performed in the data-dependent mode with a full survey MS scan followed by 10 MS/MS scans of the top 10 precursor ions from the scan. The mass spectrometer was operated in full scan mode (*m*/*z* 350-1,600) in the Orbitrap analyser at a resolution of 70,000. Data-dependent MS/MS acquisitions were performed with a 2 *m*/*z* isolation window, 27% NCE (normalized collision energy), and 17,500 resolving power.

### Data analysis and identification of putative interactors

Raw data were analysed with the Proteome Discoverer™ Software 2.2 (Thermo Fisher) using the Sequest search algorism. The Arabidopsis protein database (Araport11) was used to conduct the searches; only high confidence proteins were selected for the analysis. All peptide spectrum matches were filtered with a q-value threshold of 0.05 (5% FDR), and the proteins were filtered with high confidence threshold (0.05 q-value, 5% FDR). Nuclear proteins identified in more of two biological replicates were considered as putative interacting partners of HDA19. Localization of the proteins was gathered from published experimental evidence or–in cases where such information was unavailable–prediction inferred from the Subcellular Location of Proteins in Arabidopsis Database (SUBA).

### Gene ontology

Gene Ontology (GO) analysis was conducted using the AgriGO v2.0 toolkit web-server (Tian et al., 2017). Significantly enriched GO categories were visualised using REVIGO (Supek et al., 2011) as previously described (Vélez-Bermúdez and Schmidt, 2021).

### Protein-protein interaction network

The PPI network was constructed using STRING (https://string-db.org). Only nucleus-located partners of HDA19 (as listed in **Table 1**) were considered.

**Table 1:** Putative nuclear-localized binding partners of HDA19.

TAIR accession number	MW (kDa)	Description	Function
At5g58230	48.2	MSI1; MULTICOPY SUPRESSOR OF IRA1	Chromatin remodelling
At3g42170	78.8	DAYSLEEPER	Post-embryonic development
At1g06760	28.9	H1.1; histone 1.1	Nucleosome assembly
At3g48750	34	CDC2; CELL DIVISION CONTROL 2	DNA endoreduplication
At1g74560	30.6	NRP1; NAP1-related protein 1	Nucleosome assembly
At1g43190	48.2	PTB3; POLYPYRIMIDINE TRACT-BINDING PROTEIN 3	Regulation of RNA splicing
At3g50670	50.4	U1SNRNP; U1 SMALL NUCLEAR RIBONUCLEOPROTEIN-70K	mRNA splicing
At1g80930	104	MIF4G domain-containing protein	mRNA splicing
At3g63130	58.8	RANGAP1; RAN GTPASE ACTIVATING PROTEIN 1	Protein import into nucleus
At5g63550	59.5	DEK domain-containing chromatin-associated protein	Chromatin remodelling
At3g18790	35.4	Pre-mRNA-splicing factor ISY1-like protein	Generation of catalytic spliceosome for second transesterification step
At1g17720	56.2	ATB BETA; protein phosphatase 2A	Cell differentiation
At3g29310	61.5	Calmodulin-binding protein-related	Protein folding
At5g67630	52.1	ISE4	Chromatin remodelling
At5g55920	76.7	TRM4C, TRNA METHYLTRANSFERASE 4C	Maturation of LSU-rRNA
At1g33240	74.2	GTL1; GT-2-LIKE 1	DNA-templated transcription
At4g00238	38.2	ATSTKL1	DNA-templated transcription
At5g41340	21.3	Ubiquitin conjugating enzyme 4	Protein polyubiquitination
At5g18200	39	UTP:galactose-1-phosphate uridylyltransferases;ribose-5-phosphate adenylyltransferases	Galactose catabolic process
At5g09850	40	MED26C; MEDIATOR 26C	Transcriptional initiation
At2g19570	32.6	CDA1; CYTIDINE DEAMINASE 1	Cytidine deamination
At1g03350	51.9	BSD domain-containing protein	Unknown
At2g42810	60.2	PP5; PROTEIN PHOSPHATASE 5	Chloroplast-nucleus signalling
At1g55380	75.5	Cysteine/histidine-rich C1 domain family protein	Unknown
At2g22310	43.5	UBP4; UBIQUITIN-SPECIFIC PROTEASE 4	Protein deubiquination
At4g32270	27.3	Ubiquitin-like superfamily protein	mRNA splicing
At3g11330	55.5	PIRL9; PLANT INTRACELLULAR RAS GROUP-RELATED LRR 9	Unknown
At2g29570	29.2	PCNA2; PROLIFERATING CELL NUCLEAR ANTIGEN 2	DNA repair
At5g17020	123.2	XPO1A; EXPORTIN 1A	Protein export from nucleus
At1g02090	29.5	COP15; CONSTITUTIVE PHOTOMORPHOGENIC 15	Photomorphogenesis
At1g73030	22.7	CHMP1A; CHARGED MULTIVESICULAR BODY PROTEIN/CHROMATIN MODIFYING PROTEIN1A	Embryo development
At5g06460	119.5	UBA 2; UBIQUITIN ACTIVATING ENZYME 2	Protein ubiquitination; DNA damage response
At1g20960	247	BRR2A	mRNA processing
At1g02080	269.7	NOT1	Nuclear-transcribed mRNA catabolic process
At5g64130	15.1	cAMP-regulated phosphoprotein 19-related protein	Negative regulation of protein dephosphorylation
At3g08947	96.5	ARM repeat superfamily protein	Protein import into nucleus
At1g78150	33.1	N-lysine methyltransferase	RNA metabolism
At5g06460	119.5	UBA2; UBIQUITIN ACTIVATING ENZYME 2	Protein ubiquitination; DNA damage response
At1g63660	59.3	GMP synthase	GMP biosynthetic process
At5g06600	130.5	UBP12; UBIQUITIN-SPECIFIC PROTEASE 12	Protein deubiquitination
At3g29075	34.4	Glycine-rich protein	Regulation of gene expression
At5g40340	114.2	PDP3, PWWP DOMAIN PROTEIN 3	Epigenetic regulation of gene expression
At4g39200	12	ES25W, RIBOSOMAL PROTEIN ES25W	mRNA binding
At1g48410	116.4	AGO1; ARGONAUTE 1	Regulatory ncRNA-mediated post-transcriptional gene silencing
At3g01320	156.1	SNL1; SIN3-LIKE 1	Negative regulation of transcription
At1g67230	129	LINC1; LITTLE NUCLEI 1	Nucleus organisation
At4g38130	56	HDA1; HDA19; HISTONE DEACETYLASE 19	Histone deacetylation
At1g59890	132.9	SNL5; SIN3-LIKE 5	Negative regulation of transcription
At1g70060	150.7	SNL4; SIN3-LIKE 4	Negative regulation of transcription
At5g15020	155.1	SNL2; SIN3-LIKE 2	Negative regulation of transcription
At1g24190	151.1	SNL3; SIN3-like 3	Negative regulation of transcription
At5g08450	104.1	HDC1, HISTONE DEACETYLATION COMPLEX 1	Histone deacetylation

Localization of the proteins was gathered from published experimental evidence or prediction inferred from the Subcellular Location of Proteins in Arabidopsis Database (SUBA).

### Dataset description

**Figure 1:**
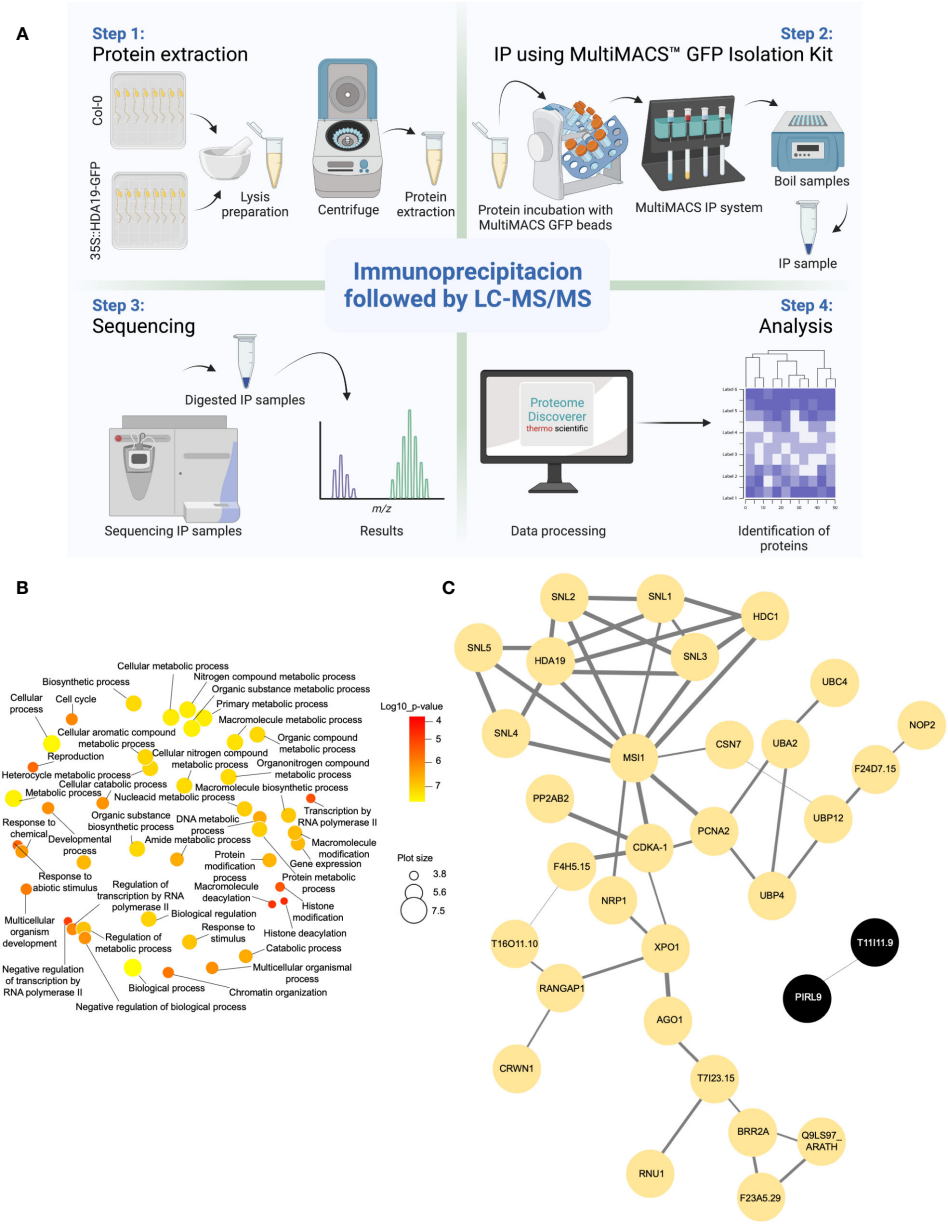
Identification of HDA19-interacting proteins. **(A)** Experimental flow of the immunoprecipitation analysis. **(B)** Overrepresented GO categories of putative nuclear-localised HDA19 interactors. Plot color indicates the log10 *P* value of enrichment, the size indicates the frequency of the GO term in the underlying GO annotation database (plots of more general terms are larger). **(C)** PPI network of putative nuclear-localised HDA19 interactors.

The identification of HDA19 protein partners via IP-LC-MS/MS relies on the capacity to distinguish true interactors from non-specific binders. To produce the current IP dataset, we used a powerful system to reduce the background in the IP samples. Samples from *Arabidopsis thaliana* Col-0 wild-type and *35S::HDA19-GFP* plants were immunoprecipitated using the MultiMACS GFP isolation kit system with µMACS MicroBeads conjugated to an anti-GFP monoclonal antibody for faster and effective magnetic labelling of GFP-tagged fusion proteins. The complete procedure is depicted in **Figure 1A**. The IP samples were digested, subjected to LC-MS/MS analysis, and HDA19-binding proteins were identified using the Proteome Discoverer software. The dataset provided a total of 371 putative interactors that were identified with high confidence (**Supplementary Data File 1**). Please note that the proteins identified in the IP using Col-0 (present in the deposited data set) were removed as background. Considering only candidate proteins that are preferentially or exclusively located to the nucleus according to experimental results or predictions, a subset of 52 putatively interacting proteins was identified in at least two biological replicates (**Table 1**). A GO enrichment analysis of this subset revealed that besides predicted processes such as ‘histone modification’, ‘chromatin organization’, and ‘negative regulation of transcription’, proteins in the categories ‘multicellular organism development’, ‘cell cycle’, ‘protein modification’ and ‘reproduction’ were overrepresented (**Figure 1B**).

A protein-protein interaction (PPI) network constructed from this subset of proteins shows a suite of well-known partners of HDA19, including five members of the SIN-LIKE (SNL) family. SNL proteins were shown to be involved in the repression of AP2 family transcription factors that repress *FLOWERING LOCUS T* (*FT*) expression through histone deacetylation (**Figure 1C**) (Huang et al., 2019; Jing et al., 2021). We also identified HDC1, a component of histone deacetylase complexes that interacts with HDA6 and HDA19 (Perrella et al., 2016). A bimolecular fluorescence complementation approach revealed that HDC1 binds to the linker histone H1 (Perrella et al., 2016), which was identified as a putative interactor of HDA19 in the current dataset. The WD-40 repeat containing protein MULTICOPY SUPRESSOR OF IRA1 (MSI1), a conserved subunit of Polycomb Repressive Complex 2 (Xu et al., 2022), and PROLIFERATING CELL NUCLEAR ANTIGEN 2 (PCNA2), involved in DNA replication and damage repair (Xue et al., 2015) were identified as central nodes of the PPI network (**Figure 1C**).

The current dataset identified a large suite of putative novel interacting partners of a key regulator of plant development and stress responses, HDA19. The identification of SNL members, HDC1, and histone H1 can be considered as validation of the current IP assay. A surprisingly large subset of (predicted) non-nuclear proteins was identified with high confidence, suggesting that some of these proteins may transiently associate with chromatin. Besides expected binding partners such as HCD1, H1, and SNLs, we found that HDA19 interacts with proteins involved in chromatin remodelling, nuclear protein export/import, protein ubiquitination associated with DNA damage repair, and chloroplast-nucleus signalling, suggesting a wide range of largely unexplored functions of HDA19 in etiolated Arabidopsis seedlings.

## Data availability statement

The mass spectrometry proteomics data have been deposited to the ProteomeXchange Consortium via the PRIDE (Perez-Riverol et al., 2016; Perez-Riverol et al., 2022; Deutsch et al., 2023) partner repository with the dataset identifier PXD045454 and can be accessed through the following link: http://www.ebi.ac.uk/pride/archive/projects/PXD045454.

## Author contributions

WS: Conceptualization, Funding acquisition, Supervision, Writing – original draft, Writing – review & editing. IV: Conceptualization, Data curation, Formal Analysis, Methodology, Visualization, Writing – review & editing.

## Supplementary material

Supplementary Data Sheet 1 Putative identified interaction partners of HDA19.
